# Epithelial-mesenchymal transition plasticity in breast cancer: regulatory mechanisms and therapeutic implications

**DOI:** 10.3389/fonc.2026.1959152

**Published:** 2026-10-14

**Authors:** Chunlei Wang, Jie Cao, Bo Qin

**Affiliations:** 1 Shaoxing Maternity and Child Health Care Hospital, Shaoxing, China; 2 Maternity and Child Health Care Affiliated Hospital, Shaoxing University, Shaoxing, China

**Keywords:** anticancer therapy, breast cancer (BC), epithelial-mesenchymal transition (EMT), regulatory mechanisms, therapeutic targets

## Abstract

Metastatic dissemination and therapeutic failure, the major determinants of breast cancer mortality, are fundamentally driven by epithelial-mesenchymal transition (EMT) and its phenotypic plasticity. This review comprehensively interrogates the regulatory logic of EMT plasticity in breast cancer, decoding the intricate interplay among transcription factor cascades, epigenetic landscapes, non-coding RNA circuits, and microenvironmental cues that collectively sustain tumor cell adaptability. We further delineate the translational frontier, summarizing recent advances in pharmacologically targeting EMT core regulators and discussing the promise and pitfalls of plasticity-modulating therapeutic paradigms. Ultimately, by integrating evolving knowledge from basic and translational research, we present a cohesive framework aimed at bridging mechanistic understanding with clinical decision-making, with the goal of refining precision therapy for breast cancer patients.

## 1 Introduction

Breast cancer (BC) is characterized by remarkable heterogeneity and a high propensity for metastasis, which remains the leading cause of mortality in affected patients. Approximately 90% of BC-related deaths are attributed to metastatic dissemination rather than the primary tumor burden, underscoring the critical need to understand the underlying mechanisms driving metastasis (1, 2). Central to this metastatic cascade is the epithelial-to-mesenchymal transition (EMT), a biological program whereby epithelial carcinoma cells lose their characteristic cell-cell adhesion and polarity, acquiring mesenchymal traits that enhance motility and invasiveness. EMT facilitates tumor cell dissemination from the primary site, enabling invasion into surrounding stroma and entry into the circulation (1). However, EMT is not a binary, irreversible event, rather, it represents a dynamic and reversible spectrum of phenotypic states, often described as epithelial-mesenchymal plasticity (EMP), which allows tumor cells to adapt to diverse microenvironmental cues and to undergo mesenchymal-to-epithelial transition (MET) at distant metastatic sites, promoting colonization and outgrowth (3). The reversible nature of EMT/MET underscores the complexity of tumor cell phenotypic modulation during metastasis and highlights the importance of understanding the regulatory networks governing this plasticity.

Recent advances have elucidated the multifaceted regulatory mechanisms orchestrating EMP in BC. Key transcription factors such as Snail, ZEB1, and Twist serve as master regulators of EMT by repressing epithelial markers like E-cadherin and inducing mesenchymal genes, thereby driving phenotypic transitions (4–9). Beyond transcriptional control, epigenetic modifications including histone methylation and acetylation dynamically modulate EMT-associated gene expression. The histone methyltransferase EZH2 has been implicated in promoting EMT through trimethylation of histone H3 lysine 27 (H3K27me3), facilitating repression of epithelial genes (10). Moreover, protein arginine methyltransferase 7 (PRMT7) induces EMT and metastasis by altering histone methylation patterns at the E-cadherin promoter, linking epigenetic reprogramming to EMT induction (11). Metabolic reprogramming also intersects with EMT regulation; alterations in oxidative metabolism and glycolytic pathways influence EMT states and tumor aggressiveness. Estrogen-related receptor gamma (ERRγ), for example, promotes MET and suppresses breast tumor growth by activating oxidative metabolism and upregulating E-cadherin (12). Conversely, metabolic shifts driven by collagen matrix density modulate EMT and invasive behavior in BC cells, highlighting the interplay between extracellular matrix remodeling and cellular metabolism (13).

The tumor microenvironment (TME) exerts profound influence on EMP and metastatic progression. Infiltrating immune cells, stromal fibroblasts, and extracellular matrix components collectively shape tumor behavior. Mast cells within the TME stimulate estrogen receptor activity in BC cells, promoting a luminal phenotype and influencing disease outcome (14). Disruption of prostaglandin E2 in cancer-associated fibroblasts (CAFs) promotes tumor EMT and metastasis (15). Myeloid cell-derived TGFβ signaling regulates extracellular matrix deposition via adenosine-dependent mechanisms, affecting fibroblast activation and tumor progression (16). The immune landscape is further complicated by EMT-mediated immunosuppression; mesenchymal-like carcinoma cells exhibit reduced MHC-I expression and increased PD-L1, fostering an immunosuppressive microenvironment that impairs cytotoxic T lymphocyte-mediated tumor clearance. Notably, minority populations of quasi-mesenchymal carcinoma cells can cross-protect epithelial neighbors from immune attack, contributing to resistance against immune checkpoint blockade therapies (17).

Therapeutic strategies targeting EMP and its regulatory networks have gained momentum, offering promising avenues to combat metastasis and therapy resistance. Small molecule inhibitors and natural compounds modulating Wnt/β-catenin signaling and EMT have shown potential in reversing EMT and reducing cancer stemness, thereby inhibiting tumor growth and metastasis (18, 19). Epigenetic modulators such as EZH2 inhibitors attenuate EMT-driven metastasis in metaplastic breast carcinoma models, highlighting the therapeutic value of targeting chromatin regulators (10). Moreover, targeting the glucocorticoid receptor axis may modulate phenotypic plasticity and overcome therapy resistance in BC (20). The development of nanoparticle-based delivery systems for siRNA against EMT regulators like Nogo-B receptor exemplifies innovative approaches to normalize tumor vasculature and suppress metastasis (21). Collectively, these advances underscore the translational potential of dissecting EMP mechanisms to identify novel targets and improve therapeutic outcomes in BC.

BC metastasis is intricately linked to the dynamic and reversible process of EMT, governed by a complex interplay of transcriptional, epigenetic, metabolic, and microenvironmental factors. Understanding the multidimensional regulation of EMP not only elucidates fundamental aspects of tumor biology but also unveils critical vulnerabilities amenable to targeted intervention. This review aims to comprehensively explore the regulatory networks modulating EMP in BC and to systematically summarize emerging therapeutic strategies that exploit these mechanisms, thereby advancing the frontier of anti-metastatic cancer therapy.

## 2 Complex transcriptional regulatory network of EMT in BC

### 2.1 The subtypes of BC and the characteristics of their EMP

BC is a molecularly heterogeneous disease conventionally classified into intrinsic subtypes (luminal A and luminal B), HER2-enriched, and triple-negative BC (TNBC)/basal-like BC, with the claudin-low subtype representing a distinct entity characterized by low expression of tight junction and adherens junction proteins, enrichment for EMT markers, and stem cell-like features (22). EMT in BC is now conceptualized as EMP, a dynamic and reversible spectrum encompassing epithelial, hybrid, and mesenchymal states that is deployed differentially across subtypes and shapes metastasis, immune evasion, and therapeutic resistance (3).

Luminal BC (hormone receptor^+^/HER2^-^) is predominantly epithelial, expressing cytokeratins 8/18 and E-cadherin, with generally restrained EMP. Partial EMT can nevertheless be induced, elevated p66ShcA promotes coexpression of epithelial and mesenchymal genes, including vimentin, N-cadherin, and Snai1/2, without obligate E-cadherin loss, thereby increasing plasticity and influencing therapeutic response (23). Luminal tumors show the highest reversible EMP between matched primary tumors and lymph node metastases, indicating that even relatively epithelial tumors retain dynamic state interconversion (24).

In HER2^+^ BC, EMP is closely linked to lineage identity and therapy resistance. HER2-amplified metaplastic carcinomas show that both spontaneous and TGFβ1-induced EMT epigenetically silence HER2 overexpression while HER2 amplification persists, providing direct evidence that EMT can drive a TNBC-like phenotypic conversion (25). Acquired trastuzumab resistance in PTEN-deficient HER2^+^ models is accompanied by EMT and a shift to TNBC-like features requiring distinct therapies, and EZH2 regulates epithelial lineage plasticity and EMT during HER2^+^ progression (26). Thus, HER2^+^ tumors possess substantial EMP capacity that can be unleashed under therapeutic pressure, blurring subtype boundaries (27).

TNBC exhibits the most pronounced and functionally integral EMP. EMP is required for successful TNBC metastasis, and ERK3/MAPK6 promotes progression through collective migration and EMP (28). TNBC is markedly heterogeneous, with cells frequently occupying hybrid E/M states that confer stemness and therapy resistance. Paradoxically, despite high baseline mesenchymal enrichment, matched primary and metastatic TNBC samples show the lowest reversible EMP, suggesting that TNBC cells may be locked in a mesenchymal-like state rather than actively interconverting. Distinguishing baseline mesenchymal phenotype from dynamic plasticity is therefore critical (28).

Claudin-low BC represents the far end of the epithelial plasticity spectrum, displaying constitutive mesenchymal properties, low-to-absent expression of claudins 3, 4, and 7, E-cadherin loss, and enrichment for EMT-inducing transcription factors TWIST1, ZEB1, and ZEB2 (29). These tumors are stem-like, low-proliferative, and highly immune-infiltrated, and can arise from luminal epithelial cells through oncogenic events such as p53 loss or KRAS activation (30). Their plasticity is evidenced by mesenchymal-to-epithelial reversal upon miR-200 family upregulation, which promotes a more epithelial morphology and reduces proliferation and metastasis, and by calorie restriction-mediated EMT reversal in claudin-low models (31). Claudin-low tumors therefore combine high mesenchymal commitment with retained epigenetic plasticity, contributing to resistance to conventional therapies.

### 2.2 Synergistic effect of EMT-inducing transcription factors

EMT is orchestrated by a cluster of transcription factors (TFs) that regulate the phenotypic plasticity of cancer cells, enabling them to acquire mesenchymal traits essential for invasion, metastasis, and stemness (**Figure 1**). Among them, the Snail family and the ZEB family are pivotal EMT inducers that directly repress epithelial markers, particularly E-cadherin, via binding to the promoter region of E-box. This repression disrupts cell-cell adhesion, facilitating the loss of epithelial integrity and the gain of mesenchymal characteristics, which are critical for BC progression (32). The Snail and ZEB proteins function not in isolation but in concert with other EMT-TFs such as Twist and TCF3, which form positive feedback loops that stabilize and sustain the EMT program. Twist, not only promotes EMT but also plays a crucial role in maintaining BC stem cells (CSCs), highlighting the intertwined nature of EMT and stemness (33). These feedback loops amplify EMT signals, ensuring the persistence of the mesenchymal phenotype and enhancing tumor aggressiveness.

**Figure 1:**
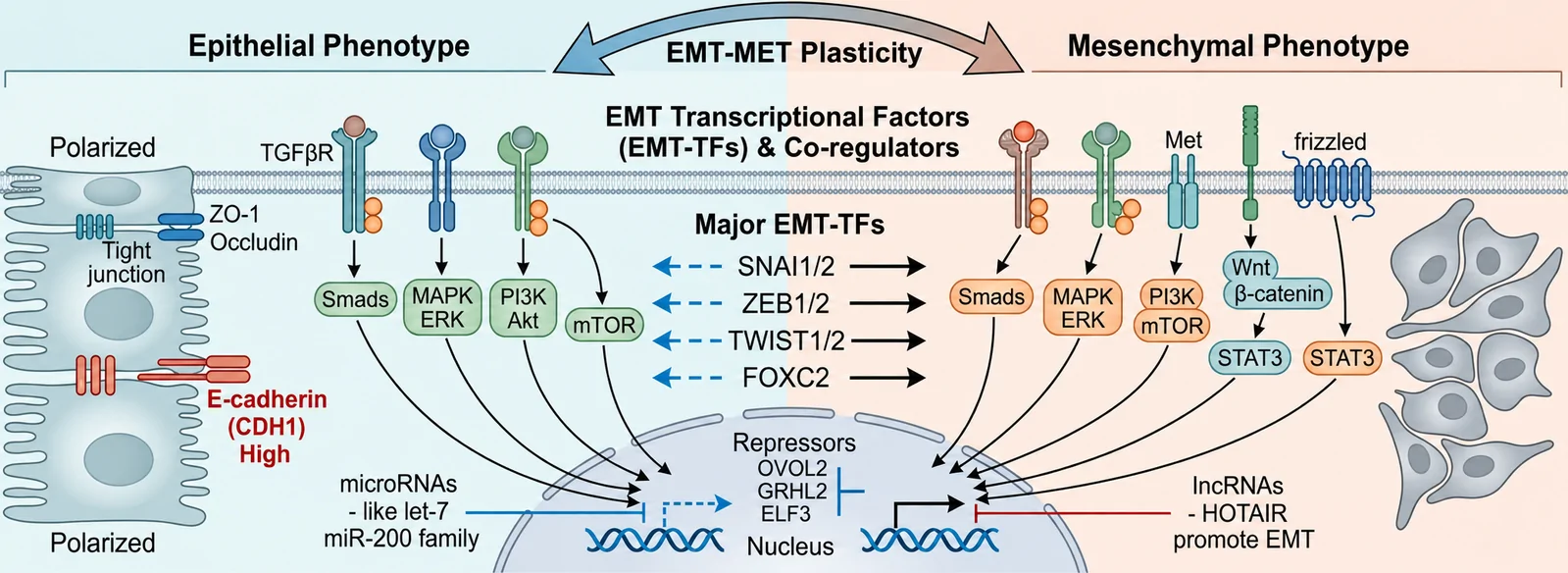
The regulatory network underlying EMP in breast cancer. EMT flexibility enables cancer cells to adapt to changing internal and external conditions during tumor progression. On the epithelial side, cells exhibit strong cell-cell adhesion, apical-basal polarity, and organized tight junctions. Key epithelial markers include CDH1, ZO-1, and occludin. EMP is controlled by several categories of upstream drivers. Mutations in PIK3CA and TP53 loss can promote EMT-related signaling. DNA methylation and histone modifications further reshape the transcriptional programs. TME provides powerful EMT-inducing signals. Smad signaling downstream of TGF-β receptors, MAPK/ERK, PI3K/AKT/mTOR, Wnt/β-catenin, and STAT3 converge in nucleus to regulate EMT-TFs and associated co-regulators, including SNAIL1/2, ZEB1/2, TWIST1/2, and FOXC2. These factors repress epithelial genes, particularly CDH1, while activating mesenchymal gene-expression programs. OVOL2, GRHL2, and ELF3 oppose EMT and help preserve or restore epithelial identity. Non-coding RNAs provide an additional regulatory layer. MicroRNAs generally inhibit EMT-promoting pathways and transcription factors. LncRNAs can promote EMT-associated gene expression.

The expression of EMT-TFs is dynamically regulated by upstream signaling pathways including TGF-β, Wnt, and Notch, which finely tune EMP (**Figure 1**). TGF-β signaling induces Snail and ZEB expression, thereby initiating EMT, while Wnt and Notch pathways further modulate the EMT-TF network to maintain the mesenchymal state and promote CSC properties (4, 6, 8, 34). Moreover, epigenetic regulators such as DOT1L operate in conjunction with c-Myc/p300-associated transcriptional complexes to epigenetically activate EMT regulators including Snail and ZEB1/2, thereby reinforcing both EMT and CSC-like traits in BC (32). Post-transcriptional regulation further amplifies this effect. PTBP3 binds the 3′UTR of ZEB1 mRNA, stabilizing the transcript and ultimately promoting EMT and metastatic spread (7).

The plasticity of EMT-TF expression is further influenced by tumor microenvironmental factors and intracellular signaling cascades (**Figure 1**). Hypoxia-inducible factors (HIFs) upregulate mitochondrial ribosomal proteins, which in turn activate ROS-Notch1-Snail signaling, linking metabolic stress to EMT induction (35). TGF-β, a master regulator of EMT and immunosuppression, promotes EMT by activating the Smad2/3 pathway, which induces mesenchymal transcription factors like Snail and ZEB1 (36). It also fosters an immunosuppressive TME by promoting M2-type macrophage differentiation and upregulating PD-L1 on cancer cells, thereby aiding immune evasion (37). TGF-β signaling in CAFs can create a positive feedback loop that enhances EMT and extracellular matrix (ECM) synthesis (38). Tumor-associated macrophages (TAMs) can secrete the ligand Wnt5a, which activates β-catenin signaling in cancer cells to induce EMT and enhance metastasis (39). CAF-derived exosomes containing microRNAs can also activate Wnt/β-catenin signaling to promote EMT (40). Notch signaling can promote EMT and stemness through Notch-Jagged signaling, which is often reinforced by inflammatory cytokines such as interleukin-6 (IL-6), it also regulates ECM components, further enhancing the invasive abilities of tumor cells (41). TNF-α, IL-6, IL-8, and IL-1β secreted by immune cells and cancer cells, activate signaling pathways such as NF-κB and JAK/STAT3 to induce EMT and promote a metastatic phenotype (5). Metabolic reprogramming in the TME profoundly influences EMT. A shift from fatty acid synthesis to fatty acid uptake is observed during EMT, a process linked to the “grow or go” switch (42). The accumulation of metabolites like succinate can also induce the transcriptional repression of EMT-related genes (43). Insulin has been identified as a direct driver of EMT in certain cancers (44). The mechanical properties of the TME, particularly ECM stiffness, are potent regulators of EMT. A stiff ECM, often resulting from increased collagen deposition by CAFs, promotes EMT through multiple signaling pathways, including FAK/SRC phosphorylation and PI3K signaling, ECM stiffness induces the expression of Snail and other EMT transcription factors and promotes the nuclear localization of TWIST1, driving invasion and metastasis (45). Phosphorylation events modulate EMT-TF activity. Akt-mediated phosphorylation of Twist1 enhances its stability and transcriptional activity, promoting TGF-β2 expression and creating a feedforward loop that sustains EMT and metastasis (8). Crosstalk between EMT-TFs and other signaling pathways further exemplifies the complexity of EMT regulation, where EMT cells can non-cell autonomously activate GLI in neighboring tumor cells to promote metastasis (46).

### 2.3 Dynamic equilibrium between EMT and MET in BC

The reciprocal interplay between EMT and MET, constitutes a central axis governing phenotypic plasticity during BC metastasis. MET enables disseminated tumor cells (DTCs) to reacquire epithelial features, which are a prerequisite for successful colonization and secondary outgrowth at distant sites (47). This cyclical EMT/MET conversion drives the metastatic cascade by conferring the adaptive flexibility required to navigate sequentially hostile microenvironments. In circulation, BC cells predominantly adopt a mesenchymal state to enhance motility and invasiveness (47). However, upon seeding at metastatic niches, they progressively revert to an epithelial phenotype via MET, a critical switch that facilitates the establishment of proliferative lesions. This reversion is tightly orchestrated by epithelial lineage activators such as GATA3 and GRHL2, which repress mesenchymal markers while simultaneously promoting epithelial differentiation (48, 49).

The hybrid states of EMT/MET often confer superior metastatic potential and therapeutic resistance compared with cells at either extreme of the phenotypic spectrum (47). Indeed, dysregulation of this dynamic equilibrium can skew metastatic behavior. Complete EMT favors dissemination but impairs colonization, whereas partial EMT optimizes both invasion and survival in adverse niches. Signaling cascades including TGF-β, Wnt/β-catenin, and receptor tyrosine kinases modulate this equilibrium by orchestrating the transcription factors and epigenetic modifiers that govern EMT and MET programs (50, 51). Pharmacological inhibition of TGF-β signaling effectively suppresses EMT and attenuates metastasis in BC models, underscoring the therapeutic relevance of targeting this plasticity axis (50, 52).

Beyond transcriptional control, the lysosomal proteolytic system, notably cysteine cathepsins, contributes to cellular remodeling during EMT and influences invasive properties independently of transcriptional reprogramming (53). Indeed, cathepsin inhibitors can reduce invasion without altering EMT gene signatures, pointing to additional layers of phenotypic regulation. Moreover, the crosstalk between EMT and CSC traits is particularly consequential, as EMT induction frequently augments stemness, thereby fueling tumor heterogeneity and treatment resilience (54). Both computational and experimental evidence indicate that EMT-associated stem cell activity is dynamically modulated by the EMT-MET cycle, reinforcing the view that plasticity is central to metastatic competence (55, 56). Collectively, the EMT/MET balance emerges as a finely tuned, multilayered regulatory network that enables efficient metastatic dissemination (47). Therapeutic strategies designed to stabilize epithelial traits, prevent dissemination, or specifically target partial EMT states to circumvent resistance therefore hold considerable promise for improving BC patient outcomes (57).

## 3 Regulatory mechanism of EMP

### 3.1 Regulatory role of epigenetic modifications in EMP

Epigenetic modifications, particularly DNA methylation and histone post-translational changes, profoundly influence EMT in breast cancer by modulating the transcriptional landscape of EMT-associated genes (58). Promoter DNA methylation, especially at CpG islands, represents a canonical mechanism of gene silencing. Hypermethylation of the *CDH1* (E-cadherin) promoter represses its transcription, a hallmark event that consolidates the mesenchymal phenotype in breast cancer cells (32, 59). The resultant loss of E-cadherin disrupts intercellular adhesion, thereby promoting cellular detachment and augmenting the migratory and invasive traits that typify EMT (60). Importantly, this epigenetic repression is reversible, underscoring the inherent plasticity of EMT and its vulnerability to therapeutic intervention. DNA methyltransferases (DNMTs), notably DNMT3B, are centrally implicated in these processes: the SNAI2-DNMT3B complex drives *ESR1* promoter methylation, leading to estrogen receptor-α (ERα) silencing and thereby facilitating EMT progression in ERα-positive breast cancers—a phenomenon associated with adverse clinical outcome (61).

Histone modifications, including acetylation and methylation, further regulate chromatin structure and gene expression during EMT. Histone deacetylases (HDACs) remove acetyl groups from histone tails, leading to chromatin condensation and transcriptional repression of epithelial markers. Concurrently, histone methyltransferases such as enhancer of zeste homolog 2 (EZH2), a component of the polycomb repressive complex 2 (PRC2), catalyze trimethylation of H3K27me3, a repressive mark that silences epithelial gene expression. These modifications act synergistically to repress epithelial genes and activate mesenchymal gene programs. For example, the recruitment of lysine-specific demethylase 1 (LSD1) by SNAI2 leads to demethylation of H3K4me2 at the ESR1 promoter, further repressing ERα expression and promoting EMT (61). Additionally, the bromodomain-containing protein 4 (BRD4) regulates EMT by modulating the transcription and stability of Snail, a key EMT transcription factor, through histone acetylation dynamics; inhibition of BRD4 reduces Snail expression and suppresses BC malignancy (4).

The reversibility of these epigenetic marks renders them attractive targets for therapeutic intervention aimed at modulating EMP. Pharmacological inhibitors of DNMTs and HDACs have demonstrated efficacy in preclinical BC models by reversing EMT-associated gene silencing and restoring epithelial characteristics. HDAC inhibitors can reactivate E-cadherin expression, thereby impairing mesenchymal traits and reducing metastatic potential. Entinostat (ENT), a histone deacetylase inhibitor, can reverses EMT of breast cancer cells by reversing the repression of E-cadherin via reducing the binding of Snail and Twist to the CDH1 promoter (62). Pracinostat, another histone deacetylase inhibitor, could suppresses BC metastasis and growth via inactivating IL-6/STAT3, showed superior anti-tumor properties compared with SAHA (63). AW01178, a novel class I HDAC inhibitor, inhibits EMT and Metastasis of BC through enhancing the acetylation level of histone H3 in the promoter region of E-cadherin (64). LBH589 (panobinostat), the pan-histone deacetylase inhibitor, induce the expression of E-cadherin on the membrane of MDA-MB-231 cells, thereby reduce cell invasion and migration (65). Trichostatin A (TSA), a specific inhibitors of HDAC1 and HDAC2, can inhibit EMT together with DNMT1 siRNA by modulating centrosome proteins γ-tubulin, TUBGCP2, pericentrin and Myc/RAS axis in TNBC cells (66). Metabolic reprogramming linked to epigenetic regulation also influences EMT plasticity. The serine, one-carbon, glycine (SOG) and methionine pathways contribute to the generation of S-adenosylmethionine (SAM), the universal methyl donor for DNA and histone methylation. Lipid metabolism-induced shifts toward these pathways, mediated by enzymes such as phosphoglycerate dehydrogenase (PHGDH), lead to epigenomic reprogramming that fosters phenotypic plasticity and EMT in breast cells (67). This metabolic-epigenetic interplay underscores the complexity of EMT regulation and suggests that targeting metabolic enzymes may indirectly modulate epigenetic states and EMT dynamics.

SWI/SNF and NuRD chromatin remodellers govern EMT by repositioning nucleosomes to control promoter accessibility of lineage-determining transcription factors (68). Through modulation of chromatin architecture, they either license or restrict expression of the EMT transcriptional program. In BC, the SWI/SNF ATPase BRG1 (SMARCA4) serves as a gatekeeper of epithelial identity, its depletion triggers EMT and confers a migratory, invasive phenotype (69). Moreover, mutations affecting BAF-complex components are enriched in aggressive triple-negative breast cancer, highlighting the clinical weight of remodeling dysfunction (70). Beyond serving as a readout of EMP, the reversible control of chromatin accessibility positions these complexes as tractable therapeutic targets. Intervening in SWI/SNF or NuRD activity could potentially restore epithelial traits, counteract metastasis, and improve patient outcomes. Chromatin remodellers therefore lie at the nexus of EMT biology and epigenetic drug discovery (68).

### 3.2 Orchestrated regulation of EMP by non-coding RNA

Long non coding RNAs (lncRNAs) and circular RNAs (circRNAs) have emerged as critical regulators of EMT in BC, a process central to tumor progression and metastasis (71). Among lncRNAs, HOTAIR and MALAT1 are the most extensively characterized. HOTAIR acts as a molecular scaffold that recruits polycomb repressive complex 2 (PRC2) to target gene loci, thereby epigenetically silencing epithelial markers and activating mesenchymal genes, a dual effect that drives EMT and augments metastatic potential. LncRNA MALAT1, in turn, modulates EMT through two principal mechanisms, sponging microRNAs (miRNAs) that target EMT inducing transcription factors, and interacting with splicing factors to influence alternative splicing events relevant to EMT. Additionally, MALAT1 engages the m6A methylation machinery via WTAP, thereby regulating hypoxia induced EMT in TNBC, which highlights an RNA modification based layer of epigenetic control (72).

CircRNAs, characterised by covalently closed loop structures, have recently been recognized as important post transcriptional regulators. Circ_0000284, for instance, promotes EMT and metastasis in TNBC by acting as a miRNA sponge, sequestering miRNAs that would otherwise suppress EMT associated gene expression. Notably, this circRNA is itself positively regulated by ZEB1, establishing a positive feedback loop that reinforces the mesenchymal state and contributes to docetaxel chemoresistance through Twist1 stabilisation. The aberrant expression of such circRNAs correlates with increased metastatic potential and adverse clinical outcomes. CircLRBA stabilize EMT transcription factors by interfering with ubiquitination pathways, thereby promoting EMT and docetaxel (DTX) chemoresistance through stabilizing Twist1. Zeb1 was reported to be able to promote the generation of circLRBA. CircLRBA was a promising biomarker and potential therapeutic target (9).

MiRNAs constitute another key regulatory tier. The miR-200 family serves as a classical suppressor of EMT by directly targeting the 3′ untranslated regions of ZEB1 and ZEB2, two potent EMT inducing transcription factors. This repression preserves E cadherin expression and epithelial integrity, and its downregulation represents a pivotal event that unleashes EMT, promoting invasion and dissemination (6). Conversely, pro metastatic miRNAs, including miR-21 and miR-10b, enhance mesenchymal traits. miR-21 promotes EMT by targeting PTEN, thereby activating downstream signaling cascades that favor mesenchymal conversion, whereas miR-10b upregulates Twist to drive cell motility and mesenchymal gene expression (73). The expression of these EMT related miRNAs is itself under multilayered control, governed by transcription factors, epigenetic modifications, and microenvironmental cues, which collectively establish intricate feedback and feed forward loops that fine tune EMT dynamics (74).

### 3.3 Regulation of EMP by TME

#### 3.3.1 Tumor-associated fibroblasts and stromal signaling

CAFs are central constituents of the BC tumor TME, exerting profound effects on malignant progression through both paracrine signaling and mechanical remodeling. A primary mechanism by which CAFs drive EMT in cancer cells involves the secretion of key soluble factors, including transforming growth factor-β (TGF-β), hepatocyte growth factor (HGF), and platelet-derived growth factor (PDGF), which activate intracellular EMT-related transcription factors, thereby conferring phenotypic plasticity that facilitates invasion and metastasis. The TGF-β pathway, in particular, is a well-established potent EMT inducer, and CAF-derived TGF-β has been shown to coordinate tumor-promoting inflammation and ECM remodeling, both of which support EMT and disease progression (75). Likewise, CAF-secreted HGF engages the c-Met axis in BC cells to stimulate proliferation and invasiveness, with matriptase identified as a critical activator of pro-HGF in stromal–epithelial crosstalk (76). PDGF signaling also plays a pivotal role in CAF activation and function; distinct CAF subsets co-expressing PDGFRβ and integrin α11 cooperate to boost tumor invasiveness via downstream JNK signaling and tenascin-C production (77).

Beyond soluble factor secretion, CAFs actively remodel the ECM by increasing collagen deposition and matrix stiffness, key biomechanical cues that stabilize EMT and potentiate metastatic dissemination. The stiffened ECM triggers integrin-mediated signaling in cancer cells, reinforcing EMT-related transcriptional programs and promoting cell motility. Elevated ECM stiffness induces mechanotransduction in mesenchymal stem cells (MSCs), driving their differentiation into CAFs that secrete prosaposin, which in turn sustains BC cell proliferation and survival (45). This bidirectional crosstalk between CAFs and the ECM establishes a feed-forward loop that perpetuates a pro-invasive milieu. CAFs drive ECM remodeling through the secretion of metalloproteinases such as ADAM10, which are packaged within exosomes and augment cancer cell motility and stemness via activation of Notch and RhoA signaling (40, 78). The dynamic ECM remodeling not only facilitates tumor cell dissemination but also generates physical barriers that impede drug delivery, highlighting the critical need to target stromal components to improve therapeutic outcomes (79).

#### 3.3.2 Role of immune cells and inflammatory signals in EMT

TME constitutes a complex ecosystem in which immune cells and inflammatory signals critically regulate EMT, a key driver of BC progression and metastasis. TAMs and myeloid-derived suppressor cells (MDSCs) represent two major myeloid populations infiltrating breast tumors, and both contribute to EMT induction and immune evasion (80). These cells secrete pro-inflammatory cytokines, including IL-6 and tumor necrosis factor-α (TNF-α), which activate key downstream signaling cascades, notably NF-κB and STAT3, within cancer cells. Activation of these pathways drives transcriptional programs that promote EMT, thereby enhancing cancer cell motility, invasiveness, and stemness, while simultaneously fostering an immunosuppressive milieu that facilitates immune escape (80). In hormone receptor-positive BC models, tumors with EMT features exhibit dense myeloid infiltration but sparse T lymphocyte presence, underscoring the immunosuppressive role of myeloid cells in sustaining EMT and tumor progression (81). Moreover, inhibition of myeloid markers such as CD11b has been shown to reduce tumor growth, reinforcing the functional importance of TAMs and MDSCs in maintaining EMT-associated aggressiveness.

Regulatory T cells (Tregs) further contribute to EMT by secreting transforming growth factor-β (TGF-β) within the TME. As a well-established EMT inducer, TGF-β directly triggers the transition of epithelial cancer cells toward a mesenchymal phenotype, characterized by loss of cell-cell adhesion and increased migratory capacity (82). Concurrently, Tregs suppress anti-tumor immune responses, thereby generating an immune-privileged niche that favors metastatic dissemination. This dual action, promoting EMT while dampening cytotoxic activity, establishes a pro-metastatic immune barrier that supports tumor cell survival and colonization at distant sites (82). Immune checkpoint molecules, particularly programmed death-ligand 1 (PD-L1), have emerged as critical modulators linking immune suppression with EMT. PD-L1 expression on tumor cells not only inhibits T-cell-mediated cytotoxicity but also engages in a positive feedback loop with EMT-related transcription factors (83). CircLRBA stabilizes the EMT-inducing transcription factor Twist1 and augments PD-L1 transcriptional activity, thereby promoting EMT, immune evasion, chemoresistance, and BC progression (9). This interplay indicates that EMT and immune checkpoint pathways are co-regulated, with PD-L1 expression potentially reinforcing mesenchymal phenotypes. Immune checkpoint inhibitors (ICIs) may reverse this immunosuppressive state and indirectly influence EMT dynamics; however, resistance to PD-1/PD-L1 blockade is frequently observed in BCs displaying pronounced EMT features, suggesting that EMT-associated immune evasion mechanisms may limit the efficacy of current immunotherapies (81). The inflammatory cytokine milieu, shaped by TAMs, MDSCs, and Tregs, orchestrates a signaling network that converges on EMT regulation. IL-6 and TNF-α activate STAT3 and NF-κB, which not only drive EMT but also upregulate immune checkpoint molecules, creating a feed-forward loop that perpetuates tumor progression and immune escape. Notably, 27-hydroxycholesterol (27HC), a cholesterol metabolite, induces EMT via STAT3 signaling, which in turn facilitates EMT and BC cell migration, illustrating the crosstalk between inflammatory signaling and EMT within the TME (84). Targeting these pathways holds potential to disrupt the pro-tumorigenic inflammatory environment and attenuate EMT-driven metastasis.

### 3.4 Interaction between metabolic remodeling and EMP

#### 3.4.1 Promotion of EMT by glucose metabolic reprogramming

During the EMT process in BC, cancer cells undergo extensive metabolic reprogramming, prominently characterized by enhanced glycolysis, a phenomenon widely recognized as the Warburg effect. This metabolic adaptation not only meets the elevated energetic and biosynthetic demands of rapidly proliferating and migrating cells, but also generates lactate and other acidifying by-products that lower the pH of the tumor microenvironment. The resulting extracellular acidification facilitates matrix metalloproteinase (MMP)-mediated degradation of ECM, thereby promoting cancer cell invasion and metastasis (85, 86). Key glycolytic enzymes, including pyruvate kinase M2 (PKM2) and hexokinase 2 (HK2), exhibit increased activity during EMT, serving not only metabolic functions but also regulatory roles in EMT transcriptional programs. PKM2 translocates into the nucleus where it interacts with β-catenin, a central mediator of Wnt signaling, to activate the transcription of Snail, a master EMT transcription factor. This nuclear function of PKM2 stabilizes and enhances Snail expression, thereby reinforcing the mesenchymal phenotype and promoting migratory and invasive capabilities of BC cells. Similarly, HK2 upregulation supports glycolytic flux and has been implicated in modulating EMT-related gene expression, further linking metabolic reprogramming to phenotypic plasticity (87).

Beyond its role as a metabolic waste product, lactate functions as a signaling molecule that remodels the epigenetic and transcriptional landscape. Histone lactylation, a recently identified post-translational modification derived from lactate, has emerged as a critical bridge between glycolytic reprogramming and gene regulation in TNBC (88). Elevated glycolysis drives lactate production, which in turn promotes lactylation at specific lysine residues, including H3K9la, H3K14la, and H3K18la, thereby enhancing chromatin accessibility and activating oncogenic transcription programs, including those governing EMT and immune modulation (89). Inhibition of glycolysis reduces histone lactylation, suppressing tumor growth and metastasis, thus highlighting the epigenetic dimension of metabolic reprogramming in EMT progression (89).

The interplay between metabolic reprogramming and EMT is further compounded by cues from TME. Hypoxia and adipocyte-derived extracellular vesicles (EVs), potentiate glycolytic activation and EMT induction. EVs from mature adipocytes promote BC malignancy by inducing hypoxia-inducible factor 1-alpha (HIF-1α) activity, which upregulates glycolytic enzymes and EMT markers, thereby facilitating invasion and metastasis. These EV-mediated effects are abrogated by HIF-1α inhibition, indicating that metabolic crosstalk within the TME reinforces EMT through glycolysis-dependent pathways (90).

In addition to direct metabolic effects, glucose metabolism influences EMT via non-coding RNAs and signaling cascades. The long non-coding RNA LINC01094, stabilized by METTL14-mediated m6A modification, acts as a molecular scaffold that facilitates PKM2 nuclear translocation and its interaction with JMJD5, thereby promoting glycolytic reprogramming and EMT. This axis enhances HIF1-α/β-catenin signaling, further driving EMT and tumor progression. Preclinical models have shown that targeting components of this pathway exerts therapeutic efficacy (87). Moreover, hyperglycemic conditions, as observed in diabetic patients, exacerbate BC progression by upregulating mediators such as mesenteric estrogen-dependent adipogenesis (MEDAG), which suppresses AMPK phosphorylation and autophagy, thereby promoting EMT and tumorigenic behaviors. This observation underscores the clinical relevance of glucose metabolic reprogramming in EMT, particularly in the context of comorbid metabolic disorders such as diabetes (91).

#### 3.4.2 New roles of lipid and amino acid metabolism in EMT

Lipid metabolism, notably enhanced fatty acid oxidation (FAO), has emerged as a key regulator of EMT in BC (92). Dysregulated lipid metabolic pathways alter membrane biophysical properties, such as fluidity and lipid composition, that are essential for cytoskeletal remodeling and pseudopodia formation, both hallmarks of EMT. Increased FAO not only supplies energy but also provides metabolic intermediates for membrane synthesis and signaling lipid production, thereby promoting actin cytoskeleton reorganization and enhancing cell motility and invasiveness. This metabolic rewiring facilitates the epithelial-to-mesenchymal phenotypic switch, enabling cancer cells to detach, migrate and colonise distant sites (92). Moreover, lipid metabolism can modulate the activity of EMT transcription factors and key signaling cascades, including TGF-β and Wnt, via lipid-derived signals, further reinforcing EMT progression. The metabolic flexibility conferred by FAO upregulation also supports cancer cell survival under metabolic stress and hypoxia, conditions that often coincide with EMT induction and metastatic spread (93).

Similarly, amino acid metabolism, particularly glutamine utilization, plays a pivotal role in supporting EMT (94). In mammals, there are two enzymes named Glutaminase (GLS1) and Glutaminase 2 (GLS2) which can catalyze the conversion of glutamine to glutamate. GLS1 is frequently upregulated in EMT-activated BC cells, as a result of direct regulation by KRAS and MYC, driving enhanced glutaminolysis. The latter one is usually regulated by p53, p63, and p73, and expressed mostly in the liver, brain, and pancreas (95). Glutamine serves as a critical carbon and nitrogen source for the biosynthesis of amino acids, nucleotides and other macromolecules required for rapid proliferation and phenotypic plasticity during EMT. This metabolic shift supplies precursors for nucleotide synthesis, thereby supporting DNA replication and repair during cellular state transitions. Notably, a dual inhibitor 9d targeting mTOR/GLS1 was designed and synthesized based on rapamycin and CB839, it had been shown to the effect of metabolic reprogramming regulation and antibreast cancer both *in vitro* and *in vivo*. (96). These observations underscore the reliance of EMT on glutamine metabolism and identify GLS1 as a promising therapeutic target for disrupting EMT-driven tumor progression.

Importantly, these metabolic hubs are not merely downstream consequences of EMT, rather, they actively participate in feedback circuits that govern EMT states. Lipid and amino acid metabolic pathways generate signaling molecules and epigenetic modifiers that modulate the expression and activity of EMT transcription factors (97). Metabolites derived from FAO and glutaminolysis can influence histone modifications and DNA methylation patterns, thereby altering chromatin accessibility and gene expression programs required for the maintenance of mesenchymal phenotypes. This bidirectional crosstalk between metabolism and EMT establishes a dynamic regulatory network that sustains cellular plasticity and metastatic capacity. Consequently, targeting these pathways offers a dual benefit: directly compromising the metabolic fitness of cancer cells while simultaneously disrupting the regulatory circuits that uphold EMT (98). Integrating metabolic inhibitors with conventional anti-EMT therapies may therefore improve treatment efficacy and circumvent resistance mechanisms driven by EMT-associated cancer progression (21).

## 4 The role of EMP in BC drug resistance

### 4.1 EMT and chemotherapy and endocrine therapy resistance

BC cells undergoing EMT acquire a mesenchymal phenotype characterized by enhanced motility, invasiveness, and stemness, which collectively contribute to therapeutic resistance. Chemoresistance in EMT-activated BC cells is frequently associated with upregulation of drug efflux transporters such as ATP-binding cassette (ABC) transporters and activation of survival signaling pathways including the AKT pathway. Studies have demonstrated that BC cells expressing high levels of lncRNA DLEU2 and receptor tyrosine kinase-like orphan receptor 1 (ROR1) exhibit enhanced EMT and CSC properties, leading to increased resistance to chemotherapeutic agents like paclitaxel and cisplatin. The overexpression of lncRNA DLEU2 and ROR1 promotes tumor cell proliferation, inhibits apoptosis, and dysregulates the cell cycle, thereby facilitating chemoresistance through EMT and CSC enrichment (99). Similarly, osteoprotegerin (OPG), a cytokine involved in bone homeostasis, has been shown to promote EMT and stemness in BC cells, correlating with increased resistance to cisplatin and docetaxel. OPG upregulation represses ERαexpression, linking EMT induction to endocrine therapy resistance by facilitating a transition to an ER-negative, more aggressive phenotype (100). This repression of ERα is a common mechanism by which EMT contributes to endocrine therapy resistance, as the downregulation or epigenetic silencing of ERα allows BC cells to escape hormone dependence and acquire invasive capabilities. For example, the SNAI2-DNMT3B complex mediates methylation of the ESR1 promoter, leading to ERα silencing and promotion of EMT, which correlates with poor prognosis in ER-positive BCs (61).

The partial EMT state is associated with a dynamic balance between epithelial and mesenchymal traits, enabling cancer cells to adapt to therapeutic pressures. Importantly, targeting EMT to reverse this plasticity has emerged as a promising strategy to overcome drug resistance (101). Experimental evidence suggests that reverting EMT can restore drug sensitivity, as cells in a partial EMT state can be coaxed back to an epithelial phenotype, thereby reactivating their susceptibility to chemotherapy and endocrine therapies. Inhibition of key EMT regulators such as BRD4 reduces the expression of the EMT transcription factor Snail, suppressing migration, invasion, and chemoresistance in BC cells (4). Additionally, targeting metabolic pathways linked to EMT, such as thymidylate synthase-mediated nucleotide metabolism, can attenuate EMT signatures and reduce chemoresistance in TNBC (102). The plasticity of EMT states also involves alternative splicing programs regulated by kinases like NEK2, which modulate mesenchymal splice variants contributing to invasive phenotypes and therapy resistance in TNBC (103).

### 4.2 Cross-regulation between targeted therapy resistance and EMP

Targeted therapies, including anti-HER2 agents and CDK4/6 inhibitors, have revolutionized BC treatment, particularly for HER2^+^ and hormone receptor^+^ subtypes. However, resistance to these therapies remains a major clinical challenge, often accompanied by the emergence of EMT features that confer enhanced plasticity and metastatic potential to tumor cells (104). In HER2^+^ BC, resistance to trastuzumab is frequently associated with upregulation of EMT transcription factors, which promote a mesenchymal phenotype and contribute to therapeutic failure. The phenotypic switch is not merely a bystander effect but actively participates in resistance mechanisms by altering cell signaling and survival pathways (105). A key molecular bridge linking targeted therapy resistance and EMP is the cross-activation of signaling cascades, including PI3K/AKT and NF-κB pathways, which are frequently hyperactivated in resistant cells, driving both EMT induction and survival advantages (8). In HER2^+^ BC models, enhanced fatty acid uptake mediated by CD36 is enriched in mesenchymal CSCs and correlates with resistance to anti-HER2 therapies. This metabolic reprogramming is regulated via Wnt signaling and contributes to the maintenance of a therapy-resistant mesenchymal CSC state. Importantly, dual blockade of CD36 and HER2 not only inhibits tumor growth but also promotes a MET-like state, restoring sensitivity to targeted agents (104). Similarly, in hormone receptor-positive BC, resistance to CDK4/6 inhibitors and mTOR inhibitors such as everolimus is linked to persistent activation of growth factor signaling pathways including IGF1R and MAPK, which are also implicated in EMT regulation. Targeting these pathways with MEK1/2 inhibitors can overcome resistance, highlighting the intertwined nature of signaling networks governing EMT and therapeutic escape (106).

Given these insights, combinatorial therapeutic strategies that simultaneously target EMP and oncogenic signaling hold promise. Preclinical studies demonstrate that combining EMT inhibitors with conventional targeted therapies enhances antitumor efficacy. HDAC6 inhibition disrupts the oncogenic functions of DCLK1, a driver of chemoresistance and metastasis, and synergizes with cisplatin in bladder cancer models, suggesting a potential parallel in BC (107). Moreover, targeting EMT-associated metabolic adaptations, such as fatty acid uptake via CD36, in conjunction with HER2 blockade, effectively reverses resistance and suppresses metastasis (104). These findings underscore the critical role of signaling pathway crosstalk in mediating the plasticity that underlies targeted therapy resistance. By disrupting the molecular circuits that sustain EMT and survival signaling, combination therapies can re-sensitize resistant BC cells and inhibit metastatic progression. Future clinical translation of these approaches requires careful patient stratification and biomarker development to identify tumors exhibiting EMT-associated resistance phenotypes.

## 5 Novel anti-BC target based on EMP

### 5.1 Screening and discovery of target molecules

High-throughput screening and systems biology methodologies have revolutionized the exploration of EMP in BC, enabling the identification of novel regulatory genes, cellular heterogeneity, and signaling pathways that govern EMT dynamics. One powerful approach is the use of CRISPR-based genome-wide knockout libraries, which facilitate functional genomic screens to pinpoint genes critical for EMP (108). Recent studies have successfully identified PRMT1 and KDM5B through CRISPR screens, highlighting their roles in modulating EMT states and suggesting their potential as therapeutic targets (109). PRMT1, a protein arginine methyltransferase, influences chromatin remodeling and gene expression, thereby impacting EMT-associated transcriptional programs. Similarly, KDM5B, a histone demethylase, modulates epigenetic landscapes that control EMP, underscoring the importance of epigenetic regulation in EMT transitions. These findings underscore the utility of CRISPR-based functional genomics in uncovering novel EMT modulators that could be exploited for targeted therapies in BC (72).

Single-cell RNA sequencing (scRNA-seq) has emerged as an indispensable tool for dissecting tumor heterogeneity and EMP at unprecedented resolution. By profiling individual tumor cells, scRNA-seq reveals distinct cellular subpopulations representing various EMT states, ranging from fully epithelial to fully mesenchymal phenotypes, as well as intermediate partial EMT (p-EMT) states (110). This granular view enables the reconstruction of cellular trajectories and transition dynamics, identifying key nodes and regulators that orchestrate EMP (110). In endocrine-resistant ER-positive BC models, scRNA-seq analysis uncovered subpopulations with elevated desmoglein-2 (DSG2) expression, which promotes phenotypic plasticity and resistance through enhanced desmosome assembly and activation of Wnt/PCP signaling. It highlights how scRNA-seq can link EMP to therapeutic resistance mechanisms and identify biomarkers predictive of poor prognosis (110). Furthermore, scRNA-seq combined with immune profiling in novel hormone receptor-positive BC mouse models has elucidated the complex TME interactions influencing EMT states and immune evasion, providing insights into resistance to immunotherapies (81).

Integrative proteomics and phosphoproteomics approaches complement genomic and transcriptomic analyses by capturing the dynamic activation states of signaling pathways during EMT. High-throughput mass spectrometry-based phosphoproteomic profiling enables the identification of kinases and signaling cascades specifically activated in EMT or p-EMT states, revealing actionable kinase targets. For instance, kinases such as AXL and ROR1 have been identified as key drivers of EMT-associated signaling networks. ROR1, a type I tyrosine kinase-like orphan receptor, is upregulated in BC cells undergoing EMT and CSC enrichment, contributing to chemoresistance, enhanced invasion, and migration. Its overexpression correlates with poor therapeutic outcomes, making it a promising kinase target for intervention (99). AXL kinase activation is linked to EMT induction and metastatic progression, and its inhibition has shown potential in reversing EMT phenotypes and sensitizing tumors to therapy. Proteomic analyses also reveal the involvement of non-receptor tyrosine kinases such as PYK2, which acts as a signaling hub integrating multiple pathways to promote EMT, stemness, and metastasis in BC, further emphasizing the complexity of kinase networks in EMT regulation (111).

The integration of multi-omics data, combining CRISPR functional screens, single-cell transcriptomics, and proteomic/phosphoproteomic profiling, facilitates a systems biology perspective on EMP. This holistic approach enables the mapping of regulatory networks and feedback loops that sustain EMT states and plasticity, identifying critical nodes amenable to therapeutic targeting. The identification of the TCF19/miR-199a-5p/SP1/LOXL2 axis through genomic and molecular analyses has revealed a novel EMT-inducing pathway that promotes BC metastasis, providing a potential target for intervention (111). Additionally, the discovery of circRNAs such as circLRBA, which stabilize EMT transcription factors like Twist1 via interaction with ubiquitin ligases, exemplifies how non-coding RNA-mediated regulation can be uncovered through integrative high-throughput approaches (9). Moreover, systems biology approaches have elucidated the role of EVs in coordinating EMT and metastatic niche formation. EVs derived from tumor cells carry molecular cargo that reprograms recipient cells and remodel distant microenvironments, facilitating EMT and pre-metastatic niche establishment. High-throughput profiling of EV contents combined with functional assays has revealed that distinct EV subpopulations synergistically regulate EMT and niche formation, suggesting novel intervention points to disrupt metastatic progression (112).

The validation of therapeutic targets involved in EMT has increasingly leveraged structural biology and medicinal chemistry approaches to design and optimize small molecule inhibitors. A prominent focus has been on disrupting protein-protein interactions (PPIs) at the interface of EMT-TFs, such as Snail and Slug, which are pivotal drivers of EMT and metastatic progression (113). Targeting these transcription factors is challenging due to their intrinsically disordered regions and the typically large, flat interaction surfaces involved in protein-DNA and protein-protein binding. Nonetheless, recent advances in structural elucidation of these complexes have enabled the rational design of small molecules that can interfere with Snail-Slug PPIs, thereby inhibiting their transcriptional activity and downstream EMT processes. These inhibitors aim to destabilize the EMT-TF complexes or prevent their binding to DNA, effectively reversing EMT and reducing metastatic potential (113, 114). However, the development of such inhibitors remains difficult due to the dynamic nature of EMT-TFs and the complexity of their interaction networks, necessitating sophisticated structural and biophysical analyses to identify druggable pockets and allosteric sites (4).

Epigenetic regulators that modulate EMT have emerged as promising targets, with several inhibitors advancing into clinical trials. EZH2, a histone methyltransferase component of the Polycomb Repressive Complex 2 (PRC2), catalyzes trimethylation of H3K27me3, leading to transcriptional repression of epithelial markers and promotion of EMT (10). The EZH2 inhibitor Tazemetostat has demonstrated potential in suppressing EMT and metastasis in preclinical models and is currently being evaluated in clinical settings for BC (27). Similarly, inhibitors targeting histone demethylases, which remove methyl groups from histone lysines and thereby regulate chromatin accessibility, have shown promise in modulating EMT-associated gene expression. These epigenetic enzyme inhibitors can reverse aberrant chromatin states that favor EMT, restoring epithelial characteristics and reducing invasiveness. The clinical progression of these agents underscores the translational potential of epigenetic modulation in EMT-targeted therapy (115). Preclinical evidence should not be presented as proof of clinical efficacy of tazemetostat in breast cancer. By contrast, HDAC inhibitors have been evaluated in randomized BC trials, including E2112, the Chinese entinostat phase III study and ACE, etc (**Table 1**).

**Table 1:** Clinical trials targeting EMT-associated pathways and therapeutic vulnerabilities in BC.

Target and treatment	Trial and population	Endpoints and key results	Safety findings	EMT connection and limits	Registry status last update
TGF-β neutralization Fresolimumab+focal radiotherapy	NCT01401062 II; randomized dose comparison Metastatic BC; n=23	Abscopal response/feasibility. Best response: SD in 3 patients; no objective responses. Higher-dose OS signal was exploratory (130).	Grade 3/4 events in 5/11 low-dose and 2/12 high-dose patients.	TGF-β/immune rationale; peripheral immune correlates. No demonstration that clinical benefit was mediated by EMT reversal.	Completed 2019-03-05
TGF-βRI/ALK5 Galunisertib+ paclitaxel	NCT02672475 I; Metastatic AR^-^ TNBC; n=29	Primary: safety/MTD. No registry results posted; no numerical efficacy result identified in this targeted search (131).	No posted safety results.	Direct pathway rationale; completed enrollment does not establish clinical anti-EMT efficacy.	Completed 2023-11-22
TGF-β trap+PD-L1 blockade Bintrafusp alfa	NCT02517398 I; TNBC expansion Pretreated advanced TNBC; n=33	Primary: confirmed response. ORR 9.1%; median PFS 1.3 months (132).	Grade 3 TRAEs: 15.2%; one treatment-related death.	Exploratory tumor HMGA2 association; not a prospectively validated EMT selection test. TNBC cohort only.	Completed 2024-05-03
TGF-β trap+PD-L1 blockade Bintrafusp alfa	NCT04489940 II; HMGA2-high TNBC; n=11	Primary: ORR. Efficacy/biomarker analyses not performed after early termination. Do not interpret as ORR 0% (133).	All-cause SAEs: 7/11.	Prospective HMGA2 selection. Sponsor judged probability of success too low; biomarker utility remains unvalidated.	Terminated 2023-04-10
AXL + PD-1 Bemcentinib (BGB324)+pembrolizumab	NCT03184558 II; Previously treated advanced TNBC; n=29	Primary: ORR. ORR 0%; DCR 3.4%; stopped after planned futility assessment (134).	All-cause SAEs: 22/29; not all attributable to treatment.	EMT-associated AXL/immune rationale; negative clinical result. Bemcentinib is a small-molecule inhibitor.	Terminated 2021-11-09
Wnt/Frizzled Vantictumab+paclitaxel	NCT01973309 Ib; Advanced HER2^-^ BC; n=48 in publication	Primary: safety/DLTs. ORR 31.3%; median PFS 3.8 months (135).	Six patients had fractures; bone toxicity limited development.	Exploratory six-gene Wnt signature; not an EMT-specific assay. No paclitaxel-only control.	Completed 2020-09-09
Notch/γ-secretase MK-0752+docetaxel	NCT00645333 I/II registration; phase I report Advanced BC; n=30	Dose finding and serial CSC assessment; decreases in CSC markers in sampled tumors (136).	DLTs included diarrhea, liver-test elevation and pneumonitis; one grade 5 pulmonary event conservatively attributed to treatment.	CD44+/CD24−, ALDH+ and mammosphere assays; CSC changes do not alone establish EMT reversal or survival benefit.	Completed 2014-04-04
Hedgehog/SMO Sonidegib+docetaxel	NCT02027376 Ib; EDALINE Advanced TNBC; n=12	Primary: MTD/RP2D. RP2D established; no DLTs observed (137, 138).	Grade 3 neutropenia and CPK elevation at highest dose.	Stromal HH/GLI1 and CSC-plasticity rationale; small exploratory translational cohort, no randomized comparator.	Completed 2023-04-03
JAK1/2–STAT3 Ruxolitinib	NCT01562873 II; pSTAT3^+^ refractory TNBC; 23 enrolled, 21 treated	Primary: ORR. No objective responses; median PFS 1.2 months (139).	Fatigue common; grade ≥3 toxicities infrequent.	pSTAT3 selection and reduced pSTAT3 in paired biopsies demonstrate pathway engagement, not clinical anti-EMT benefit.	Terminated 2017-02-23
MET/VEGFR2 and other kinases Cabozantinib	NCT01738438 II; Metastatic TNBC; n=35	Primary: ORR. ORR 9%; median PFS 2.0 months; efficacy criterion not met (140).	Fatigue, diarrhea, mucositis; dose reduction in 34%.	Exploratory soluble MET association. Multikinase activity prevents attributing effects specifically to MET or EMT.	Completed 2016-12-06
ROR1-associated stemness Zilovertamab (cirmtuzumab)+ paclitaxel	NCT02776917 Ib; Advanced HER2^-^ BC; 16 treated/analyzed	Primary: DLTs. No DLTs; PR in 6/16 (38%); median PFS 16.1 weeks (141).	Nausea attributed to zilovertamab; combination toxicity largely reflected paclitaxel.	ROR1 expression assessed in eight baseline samples; no response correlation. No randomized evidence of selective EMT-cell elimination.	Completed 2025-08-07
CXCL8/CXCR1-associated CSCs Reparixin+paclitaxel vs placebo+paclitaxel	NCT02370238 II; fRida First-line metastatic TNBC; 123 randomized	Primary: centrally reviewed PFS. 5.5 vs 5.6 months; HR 1.13, P = 0.5996 (142).	SAEs: 21.3% vs 20.0%.	ALDH+/CD24−CD44+ CSC assays; primary endpoint negative. CSC targeting is not synonymous with EMT-specific targeting.	Completed 2022-09-16
Glutaminase/GLS1 Telaglenastat (CB-839) +paclitaxel	NCT03057600 II; four cohorts Advanced TNBC; n=52	Primary: ORR. Cohorts 1–4: 22.2%, 10.0%, 10.5%, 53.8%; evaluable n=9/10/19/13 (143).	All-cause SAEs by cohort: 2/9, 2/10, 5/20, 2/13.	Indirect metabolic rationale; cohorts differ by ancestry and treatment line. No EMT enrichment or randomized control; do not pool or compare cohorts as treatment effects.	Completed 2022-09-28
HDAC/endocrine resistance Entinostat+exemestane vs placebo+ exemestane	NCT02115282 III; E2112 Advanced HR^+^/HER2^−^ BC; n=608	Co-primary PFS/OS: 3.3 vs 3.1 months (P = 0.30); 23.4 vs 21.7 months (P = 0.94) (144).	Grade 3/4 neutropenia: 20% with entinostat.	HDAC inhibition confirmed; not EMT-selected. Neither PFS nor OS improved.	Active, not recruiting 2026-08-04
HDAC/endocrine resistance Entinostat+ exemestane vs placebo +exemestane	NCT03538171 III; China Advanced HR^+^/HER2^−^ BC; 354 randomized	Primary: IRC-assessed PFS. 6.32 vs 3.72 months; adjusted HR 0.76, P = 0.046 (145).	Grade ≥3 AEs: 65.5% vs 19.3%; hematologic toxicity prominent.	Not EMT-selected; PFS benefit does not establish EMT-mediated efficacy. Population/prior-treatment differences limit comparison with E2112.	Unknown (stale registry) 2021-07-09
HDAC/endocrine resistance Tucidinostat +exemestane vs placebo+ exemestane	NCT02482753 III; ACE Postmenopausal advanced HR^+^/HER2^−^ BC; n=365	Primary PFS: 7.4 vs 3.8 months; HR 0.75, P = 0.033. Updated median OS: 30.3 months in both arms (146, 147).	Grade 3/4 neutropenia: approximately 51% in experimental arm.	Indirect epigenetic rationale; no EMT selection. PFS improvement without demonstrated OS benefit.	Completed 2022-01-12
EMT phenotype modulation Eribulin	UMIN000023300 Prospective exploratory, single arm Recurrent/metastatic BC; n=22	Primary: tissue-marker change. Epithelial/mesenchymal protein ratios increased after treatment (148).	Detailed AE frequencies unavailable in accessed abstract/registry.	Paired E-cadherin, claudin-3, vimentin and N-cadherin measurements. Direct EMT readout; no proof that marker changes caused survival benefit.	Completed 2020-07-27

The integration of artificial intelligence (AI) and computational drug discovery technologies has markedly accelerated the identification and optimization of EMT-related therapeutic candidates. AI-assisted virtual screening and molecular docking enable rapid evaluation of large chemical libraries against EMT-associated targets, including transcription factors and epigenetic enzymes (116). The in silico approaches facilitate the prediction of binding affinities, identification of novel scaffolds, and optimization of pharmacokinetic properties, thereby streamlining the drug discovery pipeline. AI-driven modeling has been instrumental in uncovering selective inhibitors that target EMT-TF PPIs or epigenetic modifiers with improved specificity and reduced off-target effects. The convergence of AI with structural biology data enhances the precision of target validation and inhibitor design, offering a promising avenue for developing next-generation EMT inhibitors with high efficacy and safety profiles (116). Future advancements in AI algorithms and integration with experimental validation are expected to yield more potent and selective agents capable of modulating EMP in BC (116, 117).

### 5.2 Targeting EMP for anti-BC therapy

In BC progression, CAF-mediated stromal signaling and glucose metabolic reprogramming represent two complementary mechanisms that drive EMT and metastasis (78, 87). Both promote invasion by remodeling the TME and ECM, albeit through distinct modes of action. Therapeutic approaches to EMT plasticity can be divided into inhibition of EMT-associated signaling, exploitation of stemness or metabolic dependencies, and modulation of cell state. **Table 1** summarizes selected BC clinical trials across these categories, including both positive and negative results. We performed a targeted narrative search of PubMed, ClinicalTrials.gov and relevant original reports, with UMIN verification for the eribulin biomarker study.

Therapeutically, interference with CAF function or their paracrine pathways offers a promising strategy to counteract microenvironment-driven EMT and tumor progression. TGF-β receptor inhibitors have proven effective in suppressing CAF-induced EMT and metastasis, underscoring the central role of TGF-β signaling in stromal-epithelial crosstalk (16). In parallel, pharmacological inhibition of Hedgehog signaling in CAFs diminishes their support of CSCs, delaying tumor formation and suggesting that CAF-directed therapies can disrupt the CSC niche (118). Reprogramming strategies, such as glycolipid-based polymeric micelles delivering angiotensin II receptor inhibitors, have been shown to reduce α-SMA-positive CAF populations, relieve stromal solid stress, decompress tumor vessels, and enhance intratumoral drug penetration, thereby producing synergistic antitumor effects (119). Furthermore, modulation of sphingosine kinase 2 (SphK2) in stromal fibroblasts has emerged as a novel approach to curb CAF activation via p53 up-regulation, reprogramming the TME to restrict BC initiation and metastasis (120). The heterogeneity of CAF populations, including bone marrow-derived CAFs that lack PDGFRα expression, carries significant implications for patient prognosis and therapeutic targeting, given that these subsets may differentially influence tumor growth and angiogenesis (121). This complexity underscores the need for precision medicine strategies that account for CAF origin and functional status. Collectively, these findings highlight the multifaceted contributions of CAFs to BC progression through EMT-inducing cytokine secretion, ECM remodeling, and reciprocal signaling with cancer cells. Targeting CAFs and their signaling networks holds considerable promise for disrupting the tumor-promoting microenvironment, inhibiting EMT, and enhancing the efficacy of combination anticancer regimens.

Pharmacological targeting of glycolysis has shown promise in reversing EMT and curbing metastatic potential in BC. Inhibitors such as 2-deoxy-D-glucose (2-DG) and PKM2-specific small molecules have been reported to suppress glycolytic reprogramming, reduce the expression of mesenchymal markers, and diminish BC cell invasiveness. Inhibition of the NQO1/PKLR axis, which modulates glycolysis via activation of AMPK and AKT/mTOR pathways, results in diminished EMT gene expression and metastatic behavior. These findings underscore the therapeutic promise of targeting metabolic enzymes to disrupt the metabolic-EMT axis in BC (85).

The development of small molecule inhibitors and biologics targeting EMT pathways represents a promising therapeutic strategy in BC, particularly given EMT’s pivotal role in tumor progression, metastasis, and drug resistance. Several small molecule inhibitors have been designed to directly or indirectly interfere with key signaling cascades involved in EMT (**Figure 2**). TGF-β receptor inhibitors such as Galunisertib have been developed to block the TGF-β signaling axis, a major inducer of EMT and tumor microenvironment remodeling. By inhibiting TGF-β receptors, Galunisertib can suppress EMT induction, thereby reducing tumor invasiveness and metastatic potential. Similarly, Wnt/β-catenin pathway inhibitors like Pripri-2 target a critical EMT regulatory axis that controls cell adhesion and transcriptional programs associated with mesenchymal phenotypes. Inhibition of this pathway disrupts the transcriptional activation of EMT-related genes, impairing cancer cell plasticity and dissemination. Additionally, NF-κB inhibitors such as Ibrutinib, originally developed as a Bruton’s tyrosine kinase (BTK) inhibitor, have demonstrated efficacy in modulating inflammatory signaling that supports EMT and tumor progression. These small molecules have shown preclinical efficacy in reducing EMT markers and metastatic behavior in BC models, highlighting their potential in clinical translation (122).

**Figure 2:**
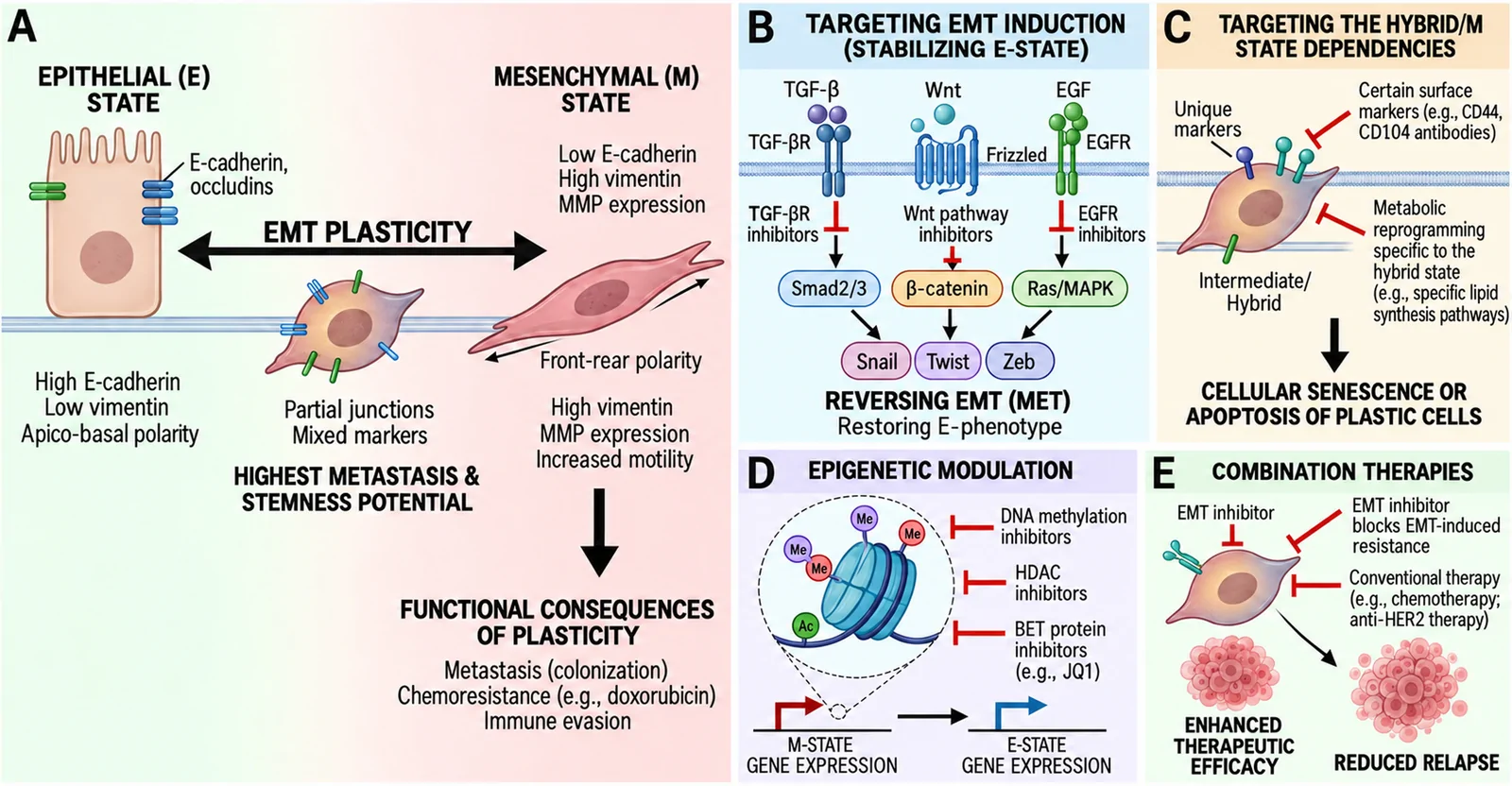
Mechanisms for targeting EMP in breast cancer therapy. **(A)** The spectrum of EMT in BC. **(B)** Targeting EMT induction for E-state stabilization. **(C)** Targeting the hybrid/M-state for anti-BC therapy. **(D)** Regulation of EMT elasticity by epigenetic drugs. **(E)** EMT inhibitor-based combination therapy for BC.

Beyond small molecules, biologics including monoclonal antibodies and bispecific antibodies have emerged as innovative tools to target EMT and the tumor microenvironment. Monoclonal antibodies against AXL receptor tyrosine kinase, such as BGB324, have been developed to inhibit AXL-mediated EMT signaling. AXL is frequently overexpressed in aggressive BCs and promotes EMT, invasion, and immune evasion. BGB324 blocks AXL activation, thereby reversing EMT phenotypes and sensitizing tumors to chemotherapy and immunotherapy. Moreover, bispecific antibodies that simultaneously target immune checkpoints and EMT-related pathways, such as anti-PD-L1/TGF-β bispecific antibodies, offer a dual mechanism to enhance antitumor immunity while inhibiting EMT-driven tumor progression. By blocking PD-L1, these agents restore T cell-mediated immune responses, and by neutralizing TGF-β, they prevent EMT induction and stromal remodeling that suppress immune infiltration. This combinatorial targeting addresses the complex interplay between EMT and immune evasion in the tumor microenvironment, potentially overcoming resistance to monotherapies (123).

Preclinical studies in BC models have demonstrated that these small molecule inhibitors and biologics can effectively suppress metastasis and reverse drug resistance associated with EMT. For example, treatment with Galunisertib and BGB324 in murine models has resulted in decreased lung metastases and improved sensitivity to endocrine and chemotherapies. Furthermore, bispecific antibodies targeting PD-L1 and TGF-β have shown enhanced infiltration of cytotoxic T lymphocytes and reduced tumor growth in immunocompetent models, underscoring the therapeutic synergy of targeting EMT and immune checkpoints concurrently (81). However, the clinical translation of these agents faces challenges due to the complexity and heterogeneity of the tumor microenvironment. The dynamic and context-dependent nature of EMT, coupled with the diverse cellular components and signaling networks within tumors, can limit the efficacy of single-agent therapies. Additionally, compensatory pathways and tumor plasticity may lead to adaptive resistance, necessitating combination strategies and biomarker-driven patient selection to optimize outcomes (52, 71, 76, 105).

Several of these agents have progressed into phase I/II clinical trials for BC, evaluating safety, pharmacodynamics, and preliminary efficacy. Early clinical data suggest manageable toxicity profiles and signs of antitumor activity, particularly in subsets of patients with aggressive or treatment-resistant disease. Nonetheless, the overall therapeutic benefit remains modest, highlighting the need for further refinement of these approaches. Advances in molecular profiling and understanding of EMT regulatory networks may enable the identification of predictive biomarkers to select patients most likely to benefit from EMT-targeted therapies. Moreover, integrating these agents with existing treatments such as endocrine therapy, chemotherapy, and ICIs may enhance efficacy by simultaneously targeting multiple facets of tumor biology (124).

### 5.3 Combination therapy guided by precision patient stratification

BC heterogeneity necessitates precise patient stratification to optimize EMT-targeted therapy. Stratifying patients by EMT-related biomarkers can identify those most likely to benefit from EMT-directed interventions (**Figure 2**). EMT signature profiling, including E-cadherin loss, vimentin/fibronectin gain, and transcription factors such as Snail, Slug, and Twist, classifies tumors along the epithelial-mesenchymal spectrum. S Aggressive subtypes, notably TNBC and claudin-low tumors, show pronounced EMT and stemness features, correlating with poor prognosis and therapeutic resistance (125, 126). EMT scores therefore separate epithelial-like, hybrid E/M, and mesenchymal-like groups. However, bulk RNA-derived EMT scores are confounded by stromal cells. Canonical mesenchymal genes, including VIM, FN1, COL1A1, COL3A1, DCN, LUM, and FAP, are abundantly expressed by fibroblasts, CAFs, endothelial cells, pericytes, and other stromal populations. A high bulk EMT score may thus reflect a large stromal compartment rather than EMT in malignant epithelial cells.

EMT should therefore be measured within malignant cells. Ideally, scRNA-seq separates malignant epithelial cells from fibroblasts and other stroma, and EMT scores are calculated only in the malignant compartment. A combined epithelial–mesenchymal framework is preferable to one or two markers: epithelial genes (CDH1/EPCAM/KRTs) versus mesenchymal genes (VIM, FN1, CDH2, ZEB1/2, SNAI1/2, TWIST1/2, and selected invasion-related genes). Such epithelial-minus-mesenchymal scoring distinguishes epithelial, hybrid, and mesenchymal malignant-cell states. When only bulk RNA-seq is available, computational deconvolution is the next-best approach. Instead of interpreting VIM/FN1 directly, one can estimate CAF/stromal abundance using COL1A1, COL1A2, DCN, LUM, COL3A1, FAP, PDGFRA, and PDGFRB with tools such as ESTIMATE, xCell, MCP-counter, EPIC, or CIBERSORTx. The EMT score can then be tested against tumor purity and fibroblast abundance, strong correlation with CAF/stromal scores suggests a microenvironment-derived signal. Across TCGA cancers, conventional EMT signatures indeed correlate positively with stromal content and negatively with tumor purity. Circulating tumor cells (CTCs) provide a minimally invasive window into EMT and metastatic potential. CTCs exhibiting EMT phenotypes, epithelial marker loss and mesenchymal marker gain, are associated with increased metastatic competency and worse outcomes (127, 128). Antibody cocktails targeting broad keratins and mesenchymal markers improve capture of mesenchymal or hybrid CTCs missed by epithelial-only isolation (128). EMT-CTC proportion and phenotype may therefore serve as predictive biomarkers for EMT-targeted therapies.

Integrating EMT biomarkers with immune-evasion and signaling features further refines stratification. EMT-high/CSC-high tumors with immunosuppressive microenvironments may require combinatorial strategies targeting EMT, stemness, and immune checkpoints (17). EMT promotes immune evasion via MHC class I downregulation, PD-L1 upregulation, and recruitment of regulatory T cells and M2 macrophages (**Figure 2**) (17), contributing to ICI resistance. TGF-β is a master regulator of EMT and immunosuppression; ALK5 inhibition suppresses EMT, reduces metastasis, and enhances immune infiltration in preclinical BC models (57). Combining TGF-β blockade with ICIs can reprogram the TME toward immunogenicity and synergize with checkpoint blockade in TNBC. Targeting EMT-associated factors such as CD73, CSF1, or SPP1 sensitizes mesenchymal-like mammary tumors to anti-CTLA-4 therapy and reverses immunosuppression (17). Mesenchymal minority cells can cross-protect epithelial neighbors from immune attack, disrupting EMT-related immunosuppressive signals dismantles this niche and amplifies ICI efficacy. TGF-β inhibitors and ICIs are generally well tolerated and co-administrable (**Figure 2**), and ongoing trials show enhanced response rates and durable remissions in EMT-high subsets. Thus, combining EMT inhibition with immune checkpoint blockade is a rational strategy to overcome immune resistance and metastasis in TNBC.

### 5.4 The development of delivery systems promotes targeted therapy

The delivery of nucleic acid-based therapeutics, including siRNA and miRNA mimics or inhibitors targeting EMT-related genes faces significant challenges, including poor stability, off-target effects, and inefficient tumor accumulation. Nanodelivery systems, such as liposomes and polymeric nanoparticles, have been developed to overcome these hurdles by enhancing the pharmacokinetics, biodistribution, and cellular uptake of RNA therapeutics in BC (129). Lipid-based nanocarriers provide biocompatible and versatile platforms for encapsulating siRNAs or miRNAs, protecting them from nuclease degradation and facilitating endosomal escape upon cellular uptake. Polymeric nanoparticles, constructed from biodegradable polymers offer tunable surface properties and controlled release kinetics (129). Surface modification with targeting ligands, such as hyaluronic acid or antibodies against tumor-associated antigens, further enhances tumor-specific delivery and cellular internalization (21).

These nanocarriers have been employed to deliver siRNAs or miRNAs that modulate EMT drivers, including transcription factors, signaling molecules, and cytoskeletal components. Redox-sensitive co-delivery micelles encapsulating salinomycin and doxorubicin demonstrated effective penetration into BC stem cell spheroids, downregulated Wnt/β-catenin and EMT-associated genes, and reversed EMT phenotypes, resulting in significant tumor growth inhibition and metastasis reduction *in vivo* (129). Similarly, tumor-acidity-responsive nanoparticles delivering siRNA against Nogo-B receptor (NgBR) normalized tumor vasculature and suppressed EMT-mediated metastasis without affecting primary tumor growth (21, 129).

Nanodelivery systems improve therapeutic safety by minimizing systemic exposure and off-target toxicity. The sustained release profiles maintain effective intracellular concentrations of RNA therapeutics, enhancing gene silencing durability. Moreover, the ability to co-deliver multiple agents enables combinatorial targeting of EMT and other oncogenic pathways, potentiating antitumor efficacy.

## 6 Conclusion

The exploration of epithelial-mesenchymal transition (EMT) plasticity in BC has unveiled a highly intricate and dynamic regulatory landscape, orchestrated by a multilayered network encompassing transcription factors, epigenetic modifications, non-coding RNAs, tumor microenvironment interactions, and metabolic reprogramming. From an expert perspective, this complexity underscores the necessity of viewing EMT not as a binary switch but as a spectrum of phenotypic states that tumor cells can traverse, thereby enabling adaptability in response to diverse microenvironmental cues and therapeutic pressures. This nuanced understanding challenges earlier simplistic models and compels the research community to integrate multifaceted regulatory axes to fully appreciate how EMP drives tumor progression, metastasis, and therapeutic resistance.

Balancing the diverse research perspectives, it is evident that while transcription factor networks such as those involving Snail, Twist, and Zeb families serve as master regulators, their activity is finely modulated by epigenetic landscapes and non-coding RNA species, including microRNAs and lncRNAs, which act as critical intermediaries in EMT regulation. Concurrently, the tumor microenvironment, through stromal cells, extracellular matrix components, and immune infiltrates, exerts reciprocal influences that can either promote or restrain EMT dynamics. Metabolic remodeling further adds a layer of complexity, linking cellular energy states and biosynthetic pathways to EMT phenotypic plasticity. Integrating these perspectives reveals a highly interconnected regulatory web rather than isolated pathways, emphasizing the importance of systems biology approaches in future investigations.

Clinically, the recognition of EMP as a pivotal driver of metastasis and drug resistance has catalyzed the identification of numerous potential therapeutic targets. Inhibitors aimed at key regulatory nodes within the EMT network, as well as combinatorial treatment strategies that simultaneously target multiple pathways, have demonstrated promising preclinical efficacy. However, translating these findings into effective clinical interventions remains challenging. Tumor heterogeneity, both inter- and intra-patient, complicates the identification of universally applicable targets. Overlapping resistance mechanisms and potential toxicities associated with targeting fundamental cellular processes necessitate cautious optimization of therapeutic windows and patient selection criteria.

To surmount these challenges, emerging technologies such as single-cell omics, patient-derived organoid models, and artificial intelligence-driven data integration offer unprecedented opportunities for precision stratification and individualized therapy design. Single-cell analyses enable the dissection of EMT states at unparalleled resolution, capturing transient and rare phenotypes that bulk analyses overlook. Organoid models faithfully recapitulate patient-specific tumor architecture and microenvironmental interactions, providing robust platforms for functional validation of therapeutic candidates. AI algorithms can synthesize complex multi-omic datasets to predict EMT dynamics and therapeutic responses, guiding rational treatment decisions.

Moreover, incorporating real-time monitoring of EMT status through minimally invasive approaches like liquid biopsy holds significant promise for dynamic clinical management. Such strategies could enable early detection of phenotypic shifts associated with metastasis or resistance, allowing timely therapeutic adjustments. Integrating EMT-targeted therapies with immunomodulatory agents and metabolic interventions could further enhance anti-metastatic efficacy by addressing the multifactorial nature of tumor progression. This holistic approach aligns with the emerging paradigm of comprehensive cancer care, aiming not only to eradicate primary tumors but also to prevent dissemination and overcome resistance.

In conclusion, the evolving landscape of BC EMP research highlights a sophisticated interplay of molecular and cellular mechanisms that govern tumor behavior. An expert synthesis of current evidence advocates for a balanced, integrative approach that reconciles diverse regulatory pathways and clinical challenges. Future progress hinges on leveraging cutting-edge technologies to achieve precise molecular characterization and personalized therapeutic strategies. By embedding EMT monitoring into clinical workflows and combining targeted, immune, and metabolic therapies, there is a tangible prospect of significantly improving patient outcomes through effective control of metastasis and resistance. This comprehensive framework sets a robust foundation for the next generation of BC research and clinical management, promising to transform EMP from a formidable obstacle into a tractable therapeutic frontier.
